# Supplementary material for: Parental haplotype-specific single-cell transcriptomics reveal incomplete epigenetic reprogramming in human female germ cells
Source: Nat Commun. 2018 May 14;9:1873. doi: 10.1038/s41467-018-04215-7 (PMC5951918; doi:10.1038/s41467-018-04215-7)
Supplement: Supplementary file 1 — Supplementary Information [file 41467_2018_4215_MOESM1_ESM.pdf]

Supplementary Information

Parental haplotype specific single-cell transcriptomics reveal incomplete epigenetic reprogramming in human female germ cells

Ábel VÉRTESY[1,2], Wibowo ARINDRARTO[3], Matthias S. ROOST[4], Björn REINIUS[5], Vanessa TORRENS-JUANEDA[4], Monika BIALECKA[4], Ioannis MOUSTAKAS[3,4], Yavuz ARIYUREK[6], Ewart KUIJK[2], Hailiang MEI[3], Rickard SANDBERG[5], Alexander VAN OUDENAARDEN[1,2,#,*] and Susana M. CHUVA DE SOUSA LOPES[4,7,#,*]

[1]Hubrecht Institute-KNAW (Royal Netherlands Academy of Arts and Sciences) and University Medical Center, 3584 CT Utrecht, The Netherlands

[2]Department of Genetics, Center for Molecular Medicine, Cancer Genomics Netherlands, University Medical Center Utrecht, The Netherlands

[3]Sequencing Analysis Support Core, Department of Biomedical Data Sciences, Leiden University Medical Center, 2333 ZC Leiden, The Netherlands

[4]Department of Anatomy and Embryology, Leiden University Medical Center, 2333 ZC Leiden, The Netherlands

[5]Department of Cell and Molecular Biology, Karolinska Institutet, 171 77 Stockholm, Sweden

[6]Leiden Genome Technology Center, Leiden University Medical Center, 2333 ZC Leiden, The Netherlands

[7]Department of Reproductive Medicine, Ghent University Hospital, 9000 Ghent, Belgium

Equal senior authorship contribution and corresponding authors

*Correspondence should be addressed to A.v.O. (a.vanoudenaarden@hubrecht.eu) or S.M.d.C.S.L. (Lopes@lumc.nl)

Table of Contents

Supplementary Figures ... **3**
Supplementary Figure 1 ... 3
Supplementary Figure 2 ... 4
Supplementary Figure 3 ... 5
Supplementary Figure 4 ... 7
Supplementary Figure 5 ... 9
Supplementary Figure 6 ... 11
Supplementary Figure 7 ... 13
Supplementary Figure 8 ... 17

Supplementary Tables ... **20**
Supplementary Table 1 ... 20

Supplementary References ... **21**

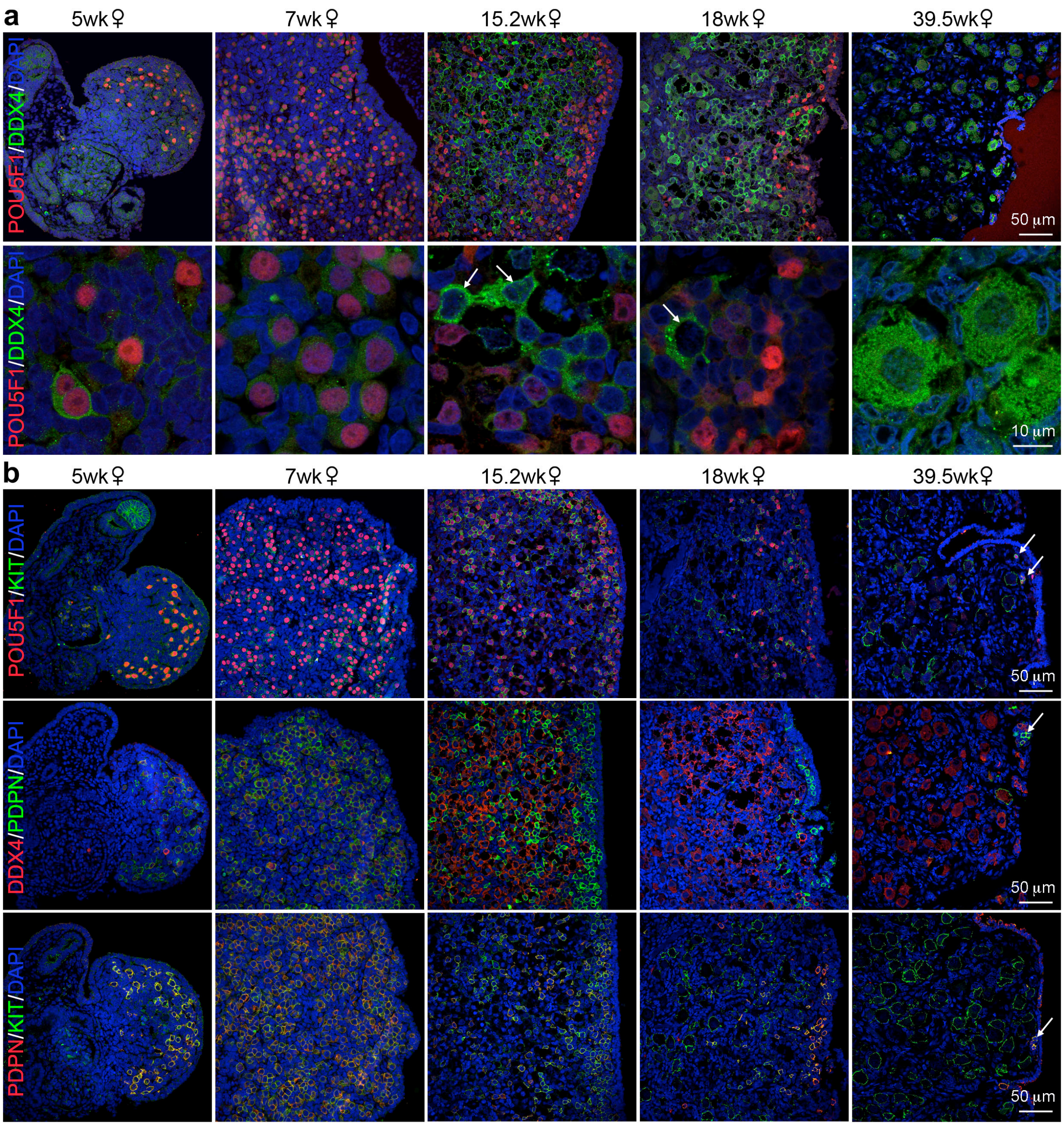

Supplementary Figure 1

Dynamic expression of germ cells markers in human female gonads from 5wk to 39.5wk.

(a) Histology sections of female gonads immunostained with *POU5F1* and *DDX4* (top row) and magnifications in the bottom row. White arrows show *DDX4*-positive germ cells that are negative for *POU5F1*. Scale bars represent 50 µm in top row and 10 µm in bottom row.

(b) Histology sections of female gonads immunostained with different combinations of *POU5F1, DDX4, KIT, PDPN*. White arrows show *POU5F1*-positive/*PDPN*-positive/*KIT*-positive germ cells at the surface of the 39.5wk. Scale bars represent 50 µm in all rows.

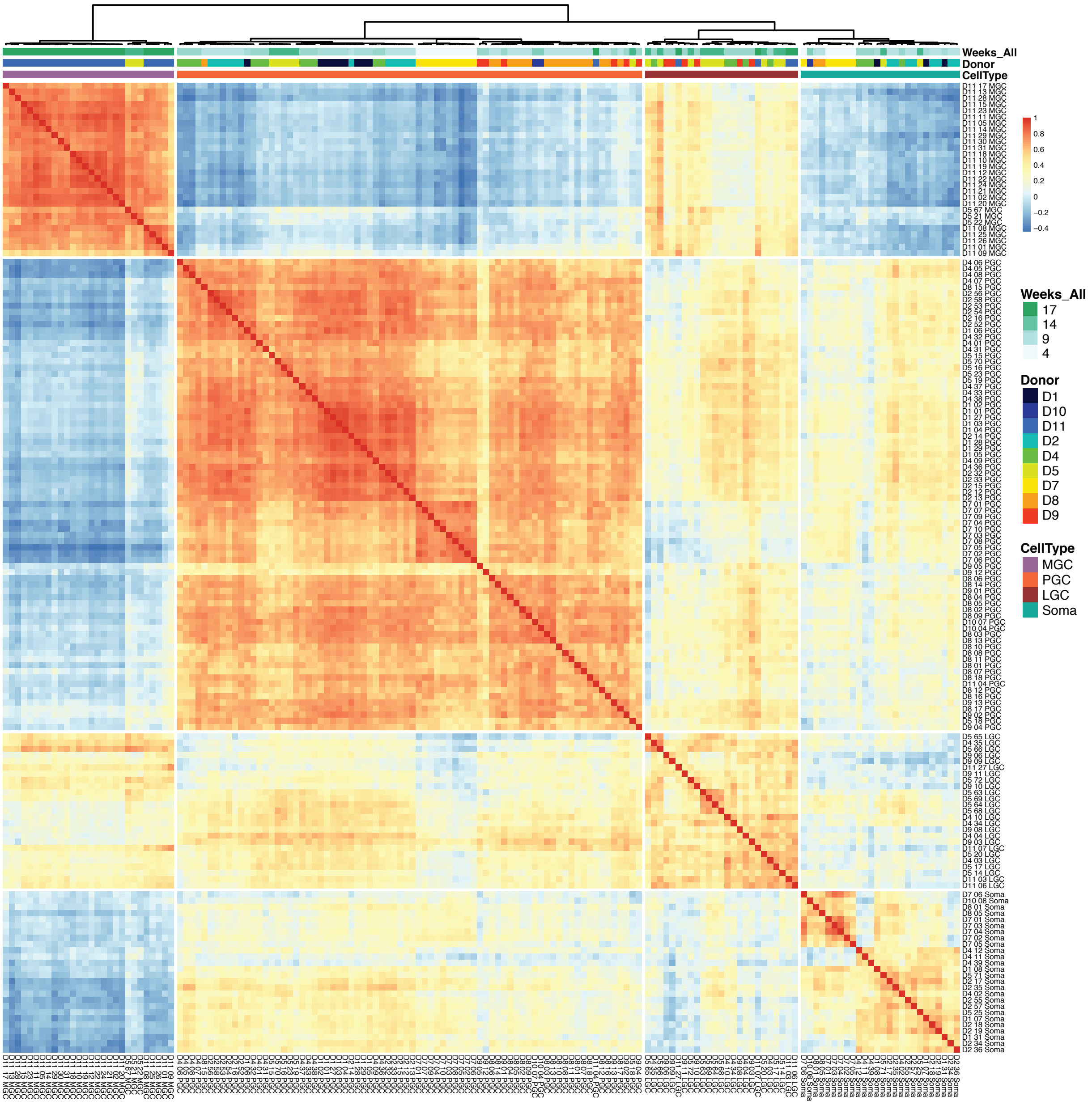

Supplementary Figure 2

Individual human female germ cells cluster by developmental stage.

Spearman correlation of single-cell transcriptomic profiles of germ cell related genes (see Fig. 2a) combining our single-cell human female dataset with the online available single-cell human female dataset from Guo et al., 2015 gave a total of 129 female germ cells and 26 female somatic cells from 9 different donors. In the top bar, each single cell is coloured by week of development, donor embryo, and cell type or developmental stage. Note that the germ cells cluster by developmental stage (PGC, LGC, MGC).

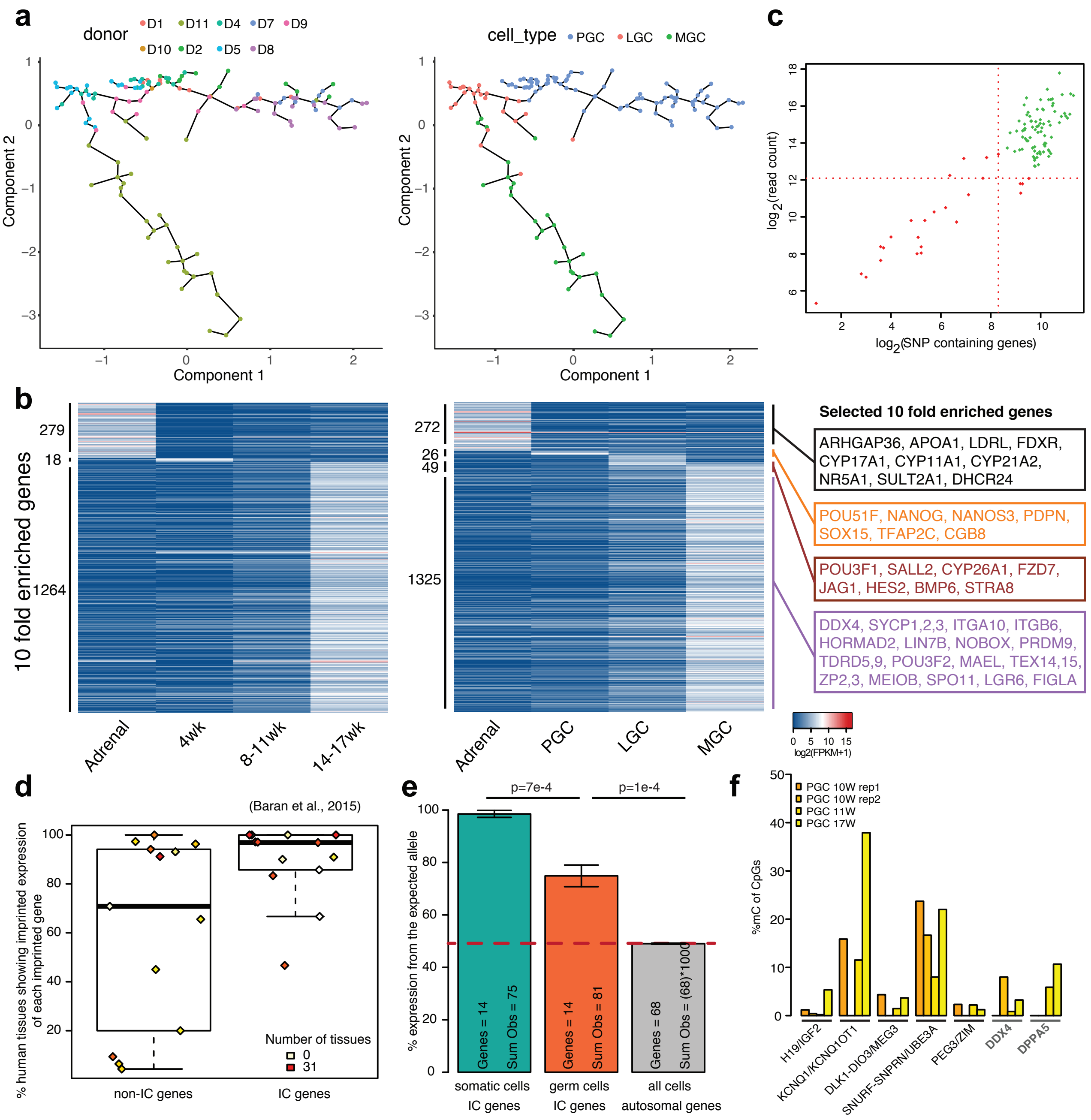

Supplementary Figure 3

Developmental characteristics of the human female germ cells.

(a) Minimal spanning tree (monocle) providing developmental trajectory of female germ cells. High quality female germ cells, combining our single-cell human female dataset with the online available single-cell human female dataset from Guo et al., 2015 gave a total of 129 female germ cells and 26 female somatic cells from 9 different donors. Pseudo-time is determined by projecting all cells on the trunk (main connecting line) and is concordant with development stage (PGC, LGC, MGC).

(b) 10-fold enrichment transcriptional signature in female adrenal and germ cells (our female germ cell dataset combined with the female germ cell dataset from Guo et al., 2015) per developmental age group (4wk, 8-11wk, 14-17wk) (left panel) and germ cell stage group [primordial germ cells (PGC), late germ cells (LGC) and meiotic germ cells (MGC)] (middle panel). The number of 10-fold enriched genes is depicted on the left side of the plots. The full list of genes in each category is given in Supplementary Data 1.
(c) Filtering single cells on total read counts and SNP-containing gene counts. To reliably compare allelic expression, cells with both high read- and SNP-containing gene count were selected (green). The cells in the lowest quintiles (lowest 20%, below or left of the dotted lines) in each dimension (cells in red) were not used for analyses.
(d) Graph depicting the percentage (%) of different human tissues showing imprinted expression for either the imprinted genes belonging to the 5 studied imprinted gene clusters (IC genes, right) or the rest (non-IC genes, left). The number of different tissues showing expression is denoted as the colour of each dot (gene) from white to red. The difference was significant (p=0.028) on a one-sided Mann-Whitney-Wilcoxon test. The dataset used was from Baran et al., 2015.
(e) Average allelic bias (fraction of expression from the expected allele) across genes belonging to the 5 studied imprinted gene clusters (IC-genes) in somatic (green) and germ cells (orange). The number of IC-genes with informative SNP was 14 and was observed (expressed) in total 75 times in somatic cells and 81 times in germ cells. As control, allelic-bias for 1000 randomly selected autosomal genes across all 68 germ cells (with allelic expression data) was calculated (grey). The average allelic bias in germ cells is significantly lower than in soma, but significantly higher than in randomly sampled autosomal genes from all cells (1000 bootstrapping rounds). Average bias with s.e.m. (error bars) is reported for each case. p-values were calculated in pairwise, one-sided Mann-Whitney-Wilcoxon tests.
(f) Methylation rate of the selected imprinting control regions separated per germ cell sample from Guo et al., 2015. As control, the promoter region of *DDX4* and *DPPA5* demethylated in germ cells is also depicted.

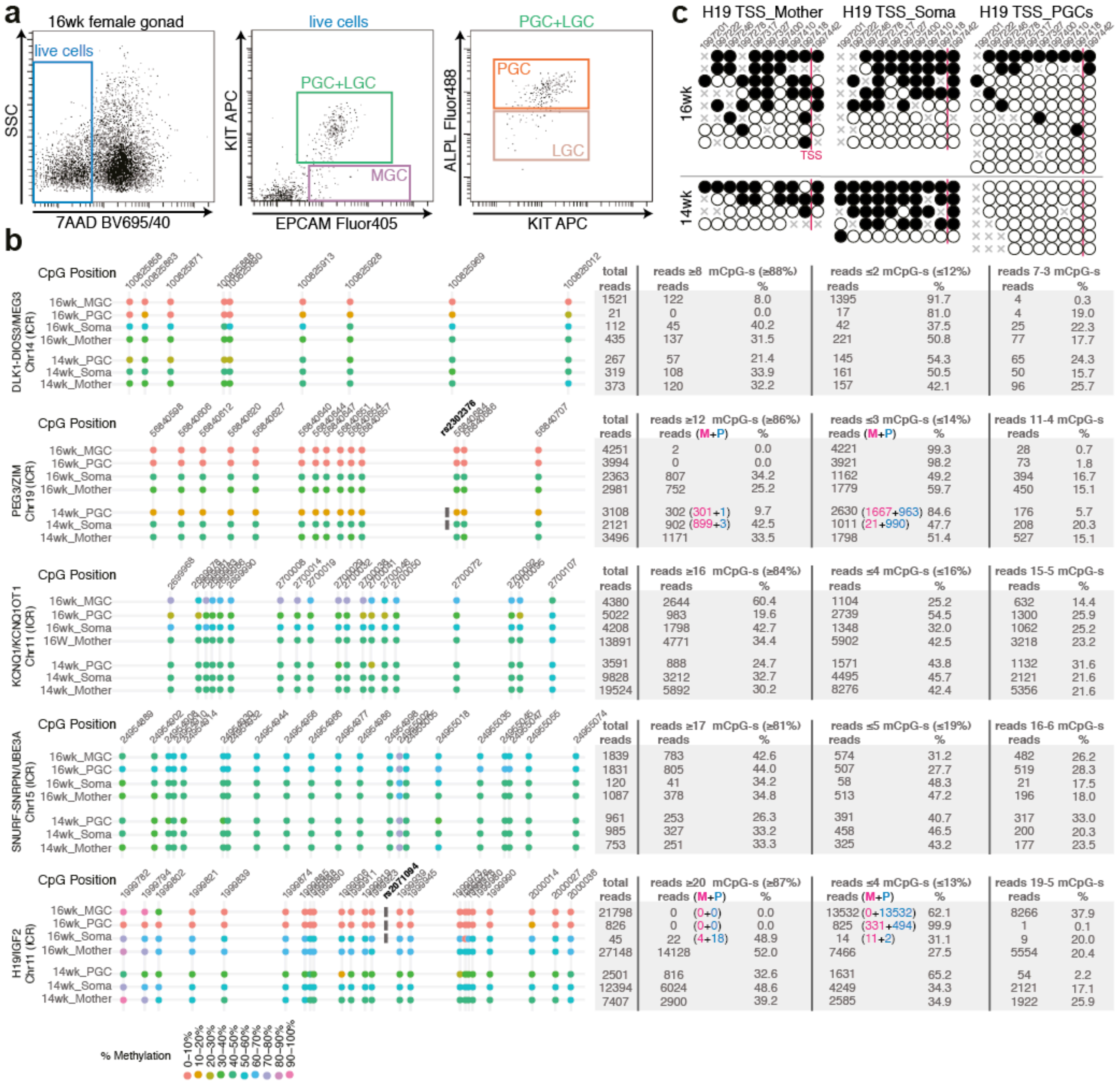

DLK1-DIOS3/MEG3 Chr14 (ICR)

Sample	total reads	reads ≥8 mCpG-s (≥88%) reads	%	reads ≤2 mCpG-s (≤12%) reads	%	reads 7-3 mCpG-s reads	%
16wk_MGC	1521	122	8.0	1395	91.7	4	0.3
16wk_PGC	21	0	0.0	17	81.0	4	19.0
16wk_Soma	112	45	40.2	42	37.5	25	22.3
16wk_Mother	435	137	31.5	221	50.8	77	17.7
14wk_PGC	267	57	21.4	145	54.3	65	24.3
14wk_Soma	319	108	33.9	161	50.5	50	15.7
14wk_Mother	373	120	32.2	157	42.1	96	25.7

PEG3/ZIM Chr19 (ICR)

Sample	total reads	reads ≥12 mCpG-s (≥86%) reads (M+P)	%	reads ≤3 mCpG-s (≤14%) reads (M+P)	%	reads 11-4 mCpG-s reads	%
16wk_MGC	4251	2	0.0	4221	99.3	28	0.7
16wk_PGC	3994	0	0.0	3921	98.2	73	1.8
16wk_Soma	2363	807	34.2	1162	49.2	394	16.7
16wk_Mother	2981	752	25.2	1779	59.7	450	15.1
14wk_PGC	3108	302 (301+1)	9.7	2630 (1667+963)	84.6	176	5.7
14wk_Soma	2121	902 (899+3)	42.5	1011 (21+990)	47.7	208	20.3
14wk_Mother	3496	1171	33.5	1798	51.4	527	15.1

KCNQ1/KCNQ1OT1 Chr11 (ICR)

Sample	total reads	reads ≥16 mCpG-s (≥84%) reads	%	reads ≤4 mCpG-s (≤16%) reads	%	reads 15-5 mCpG-s reads	%
16wk_MGC	4380	2644	60.4	1104	25.2	632	14.4
16wk_PGC	5022	983	19.6	2739	54.5	1300	25.9
16wk_Soma	4208	1798	42.7	1348	32.0	1062	25.2
16W_Mother	13891	4771	34.4	5902	42.5	3218	23.2
14wk_PGC	3591	888	24.7	1571	43.8	1132	31.6
14wk_Soma	9828	3212	32.7	4495	45.7	2121	21.6
14wk_Mother	19524	5892	30.2	8276	42.4	5356	21.6

SNURF-SNRPN/UBE3A Chr15 (ICR)

Sample	total reads	reads ≥17 mCpG-s (≥81%) reads	%	reads ≤5 mCpG-s (≤19%) reads	%	reads 16-6 mCpG-s reads	%
16wk_MGC	1839	783	42.6	574	31.2	482	26.2
16wk_PGC	1831	805	44.0	507	27.7	519	28.3
16wk_Soma	120	41	34.2	58	48.3	21	17.5
16wk_Mother	1087	378	34.8	513	47.2	196	18.0
14wk_PGC	961	253	26.3	391	40.7	317	33.0
14wk_Soma	985	327	33.2	458	46.5	200	20.3
14wk_Mother	753	251	33.3	325	43.2	177	23.5

H19/IGF2 Chr11 (ICR)

Sample	total reads	reads ≥20 mCpG-s (≥87%) reads (M+P)	%	reads ≤4 mCpG-s (≤13%) reads (M+P)	%	reads 19-5 mCpG-s reads	%
16wk_MGC	21798	0 (0+0)	0.0	13532 (0+13532)	62.1	8266	37.9
16wk_PGC	826	0 (0+0)	0.0	825 (331+494)	99.9	1	0.1
16wk_Soma	45	22 (4+18)	48.9	14 (11+2)	31.1	9	20.0
16wk_Mother	27148	14128	52.0	7466	27.5	5554	20.4
14wk_PGC	2501	816	32.6	1631	65.2	54	2.2
14wk_Soma	12394	6024	48.6	4249	34.3	2121	17.1
14wk_Mother	7407	2900	39.2	2585	34.9	1922	25.9

Supplementary Figure 4
DNA methylation of imprinting control regions in FACS-sorted human female germ cells.
(a) FACS gating strategy for the isolation of different germ cell populations identified in the 16wk female gonad. Live cells (7AAD-negative) were separated into EPCAM-negative somatic cells and EPCAM-positive germ cells. Thereafter, *KIT*-negative MGC; *KIT*-positive, *ALPL*-low LGC; and *KIT*-positive, *ALPL*-high PGC populations were sorted.
(b) Methylation ratios per CpG position along each ICR region. Coloured circles represent the average methylation rate per CpG position across all reads per sample. FACS-sorted germ cells (PGCs and MGCs), somatic, and maternal tissues of two different individuals (14wk and 16wk) were analysed. Corresponding tables on the right show total read counts, as well as read counts and fractions for reads binned into 3 categories: methylated, unmethylated, partially methylated reads. Two informative SNPs (rs2302376

and rs2071094) allowed to separate maternal (M, pink) and paternal reads (P, blue) for the H19 and *PEG3* loci in the 16wk and 14wk individual, respectively.

(c) Methylation status per CpG position in the proximal promoter region bordering with the transcriptional starting site (TSS) of *H19* in FACS-sorted PGC, somatic, and maternal tissues of two different individuals (14wk and 16wk). Each row represents a different read, white circles represent an unmethylated CpGs, black circles methylated CpGs and grey crosses represent no information at that CpG position. Red line marks the position of the TSS.

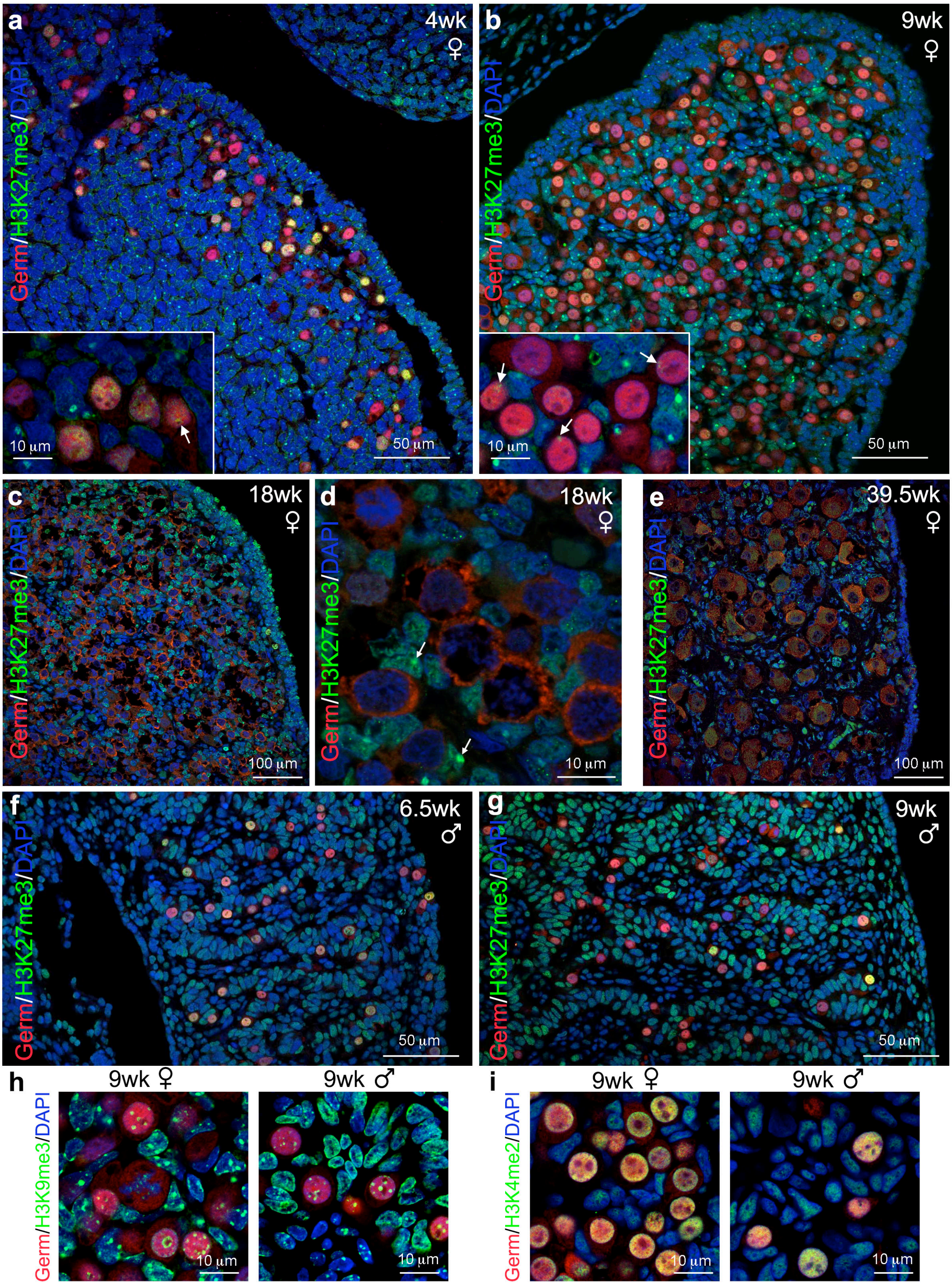

Supplementary Figure 5
Selected chromatin marks in male and female germ cells during development.

(a-e) Histological sections of 4wk (a), 9wk (b), 18wk (c, d) and 39.5wk (e) female gonads immunostained for histone H3 lysine 27 trimethylation (H3K27me3) and germ cell markers (early-marker *POU5F1* and late-marker *DDX4*) identify all germ cells (in red). Inserts in (a,b) depict magnified field and white arrows point to perinuclear H3K27me3 foci, probably corresponding to the reactivating X chromosome. White arrows in (d) point to the characteristic perinuclear H3K27me3 foci of the silent X chromosome in female somatic cells. Scale bars represent 50 µm in (a,b,f,g), and 10 µm in the inset of (a,b). Scale bars in (c,e) represent 50 µm, and 10 µm in (d, h, i).
(f-g) Histological sections of 6.5wk (f) and 9wk (g) male gonads immunostained for H3K27me3 and germ cell markers (early-marker *POU5F1* and late-marker *DDX4*) identify all germ cells (in red).
(h) Histological sections of 9wk female (left) and male (right) gonads immunostained for histone H3 lysine 9 trimethylation (H3K9me3) and germ cell markers (early-marker *POU5F1* and late-marker *DDX4*) identify all germ cells (in red).
(i) Histological sections of 9wk female (left) and male (right) gonads immunostained for histone H3 lysine 4 dimethylation (H3K4me2) and germ cell markers (early-marker *POU5F1* and late-marker *DDX4*) identify all germ cells (in red).

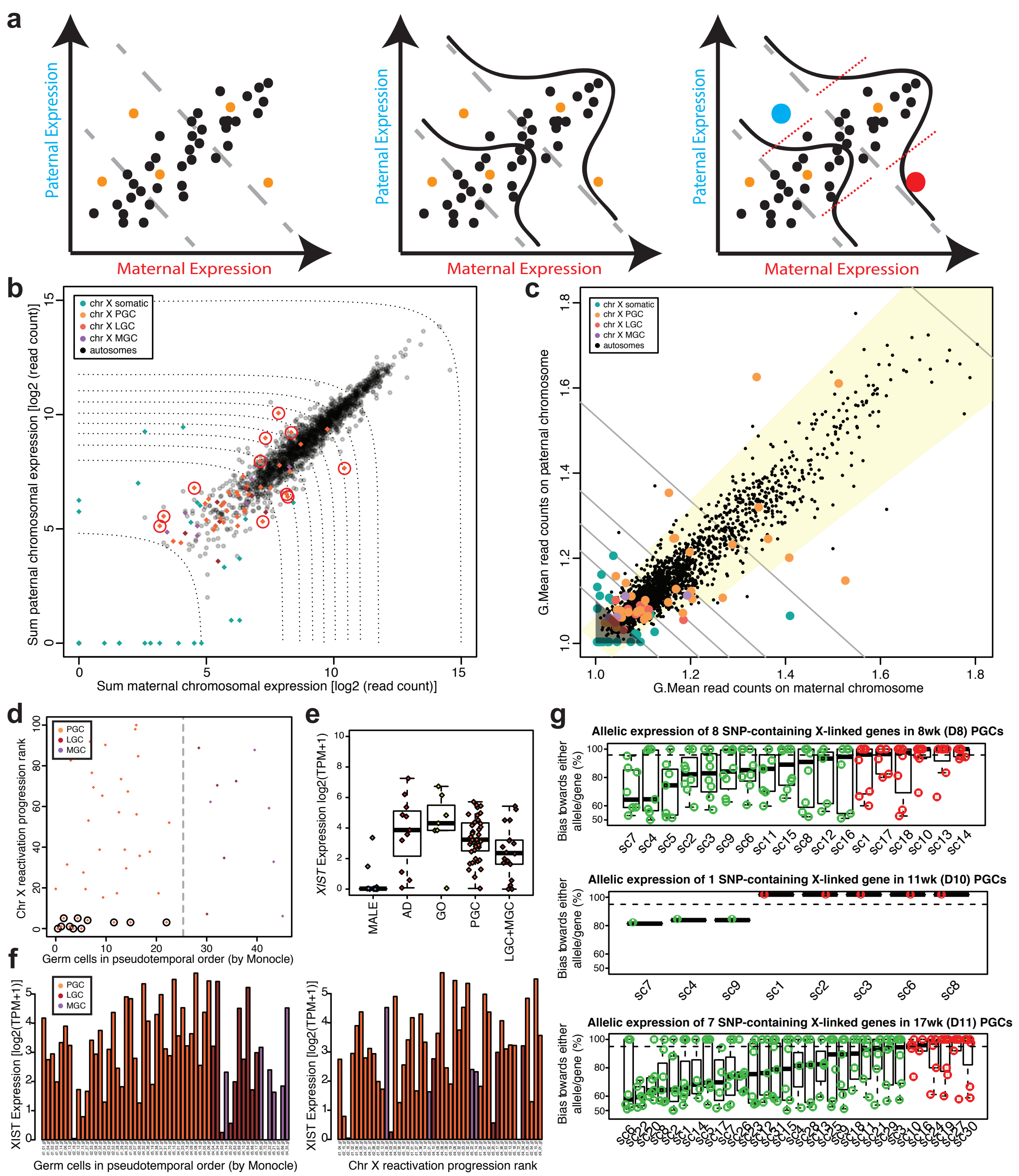

Supplementary Figure 6

XCI state of female germ cells.

(a) Cartoon depicting how Fig. 4c was generated: (left) each dot reflects the sum of paternal and maternal reads of an autosomal chromosome (black dots) or the X chromosome (orange dots) per single cell; (middle) bins were created to contain the same amount of dots per subsequent expression bins. Confidence intervals per bins were calculated so that they contain 95% of the autosomes. There are 3 categories of PGCs

(right panel): with two active X chromosomes (orange dots); with a paternally active X chromosome (blue dot) and with a maternally active X chromosome (red dot).

(b) Log-scale variant of Fig. 4c. Allelic bias of individual autosomes (black) and sex chromosomes (coloured by cell type and stage). The sum of maternal and sum of paternal allele-specific read counts of SNP-containing genes per chromosome per single cell shows different degrees of reactivation. Chromosomes are binned per total allelic read counts (dashed lines) to counter sequencing depth related technical effects. X chromosomes in orange circles are from germ cells that fall outside that 95% interval, therefore are significantly not reactivated. The read counts of the X chromosome exclude the escapees.

(c) Geometric mean based variant of Fig. 4c. When summarizing chromosomes, the geometric mean, instead of the sum of allelic reads, is calculated for both autosomes and the X chromosome. The yellow area in each bin corresponds in this case to the 99% confidence interval on autosomal chromosomes per bin, marking the boundary of highly significant allelic bias.

(d) Correlation between female germ cell ranking in pseudo-temporal order (monocle) (Fig. 2d) and the chromosome X reactivation ranking (Fig. 4e). The grey dashed line separates PGC from LGC and MGC. In black circles are the female germ cells (11 PGCs) that fell outside the confidence interval that contained 95% of the (biallelic) autosomes.

(e) *XIST* expression in single cells per cell type [somatic male cells (MALE), female adrenals cells (AD), female somatic gonadal cells (GO), primordial germ cells (PGC) and pooled late and meiotic germ cells (LGC+MGC).

(f) *XIST* expression in single female germ cells ranked in pseudo-temporal order as determined by monocle (left panel) and ranked by X chromosome reactivation progression (right panel). Germ cells are coloured by developmental stage.

(g) Systematic analysis of allelic expression bias in the dataset from Guo et al., 2015. Allelic expression bias of the all SNP-containing, non-escapee X-linked genes in 8wk (D8) embryo (8 genes, top panel), 11wk (D10) embryo (1 gene, middle panel) and 17wk (D11) embryo (7 genes, bottom panel). Each dot represents expression bias of each of the SNP-containing non-escapee X-linked genes towards one of the alleles (50% means 50:50 expression; 100% means monoallelic expression towards one allele) per PGC. There is no paternal haplotype information in this dataset and without that the full 0-100 % allelic bias spectrum cannot be reconstructed for an ensemble of genes. For genes with multiple SNPs, the average expression bias is calculated. Each embryo has several PGCs that showed a median of monoallelic ratio above 95% (marked in red): in 8wk (D8) embryo [6 out of 18 (33.3%), top panel], 11wk (D10) embryo [5 out of 8 (62.5%), middle panel] and 17wk (D11) embryo [6 out of 31 (19.4%), bottom panel].

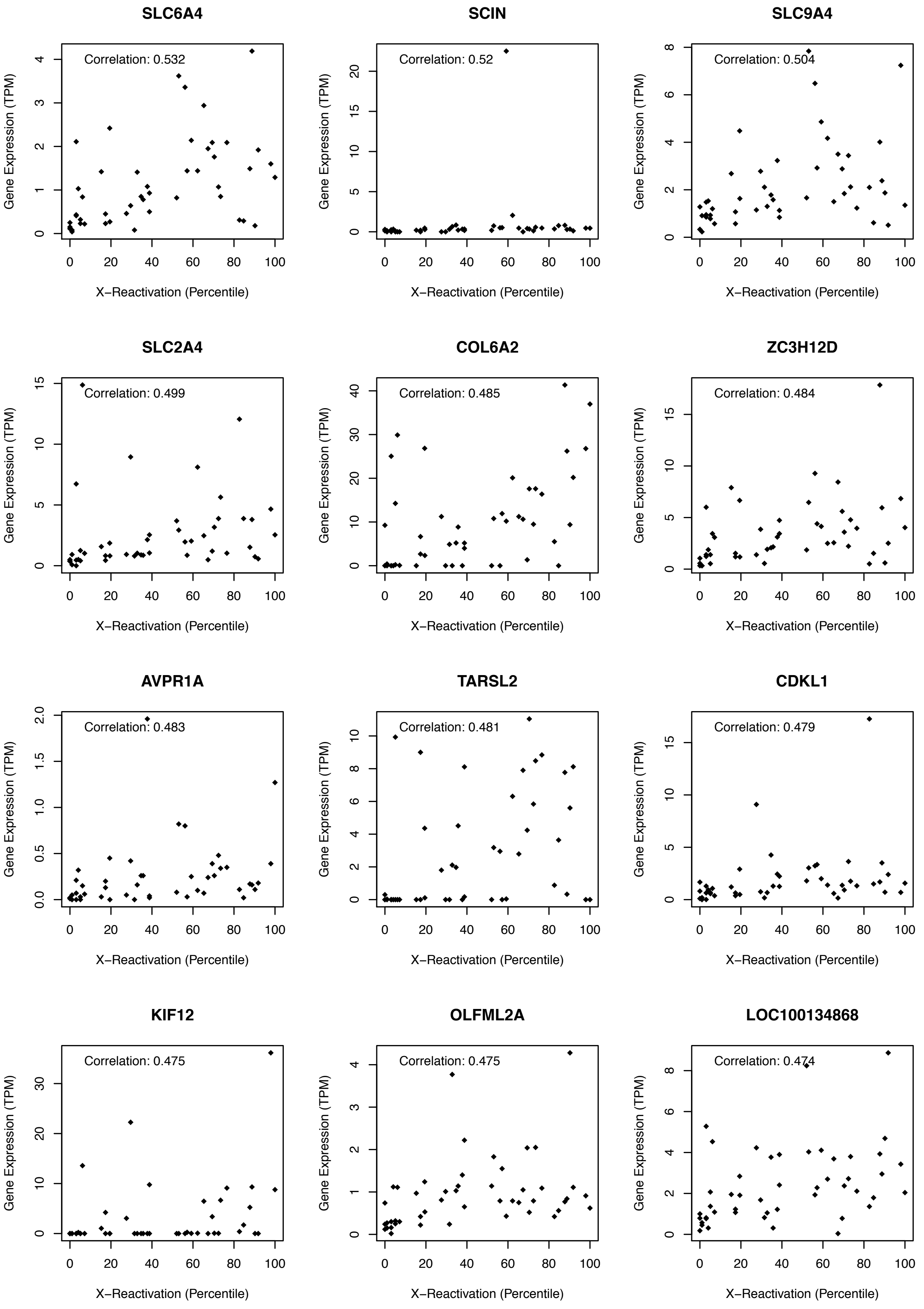
SLC6A4
Correlation: 0.532
SCIN
Correlation: 0.52
SLC9A4
Correlation: 0.504
SLC2A4
Correlation: 0.499
COL6A2
Correlation: 0.485
ZC3H12D
Correlation: 0.484
AVPR1A
Correlation: 0.483
TARSL2
Correlation: 0.481
CDKL1
Correlation: 0.479
KIF12
Correlation: 0.475
OLFML2A
Correlation: 0.475
LOC100134868
Correlation: 0.474
Gene Expression (TPM)
X−Reactivation (Percentile)

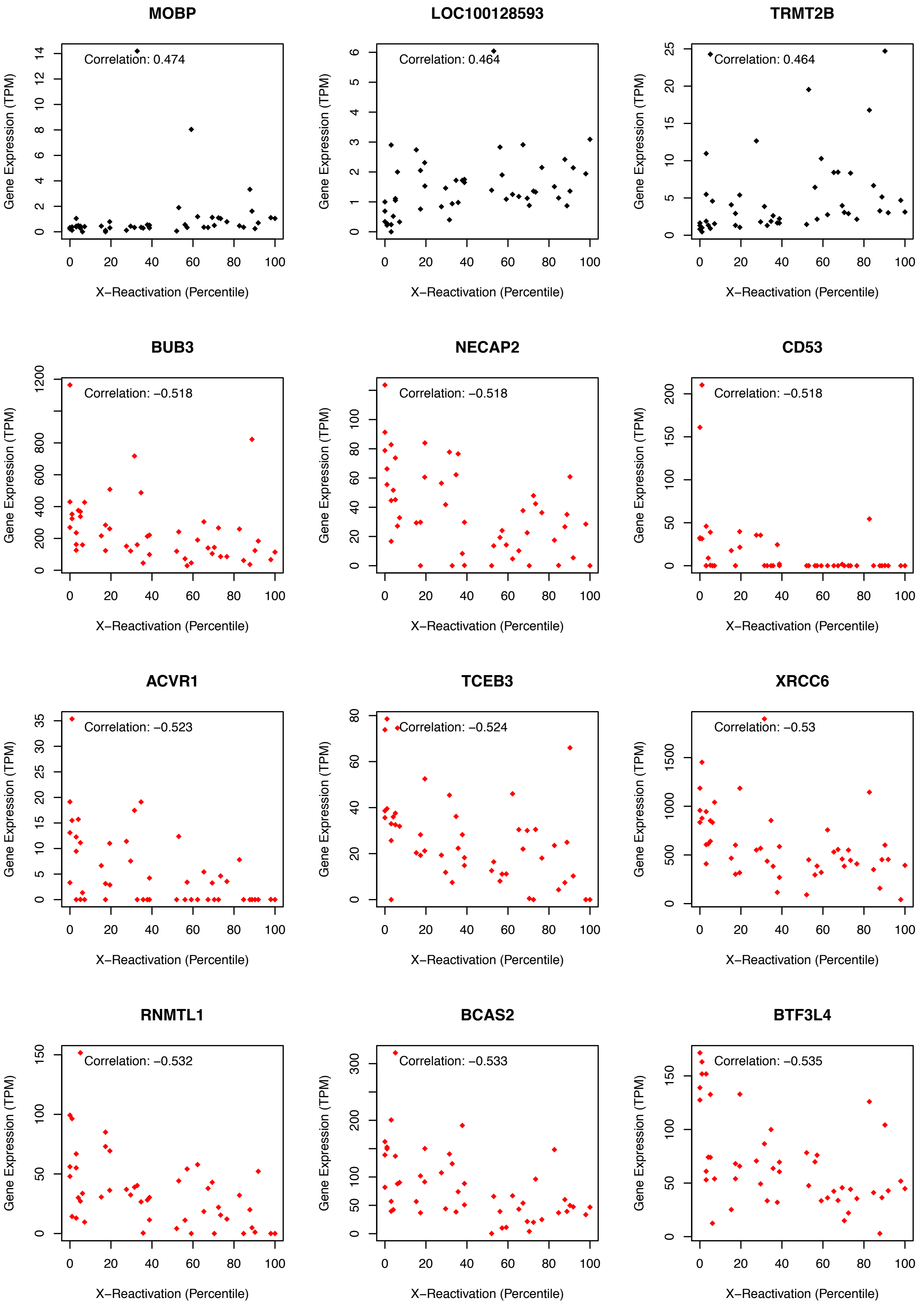
MOBP
Correlation: 0.474
LOC100128593
Correlation: 0.464
TRMT2B
Correlation: 0.464
BUB3
Correlation: −0.518
NECAP2
Correlation: −0.518
CD53
Correlation: −0.518
ACVR1
Correlation: −0.523
TCEB3
Correlation: −0.524
XRCC6
Correlation: −0.53
RNMTL1
Correlation: −0.532
BCAS2
Correlation: −0.533
BTF3L4
Correlation: −0.535
Gene Expression (TPM)
X−Reactivation (Percentile)

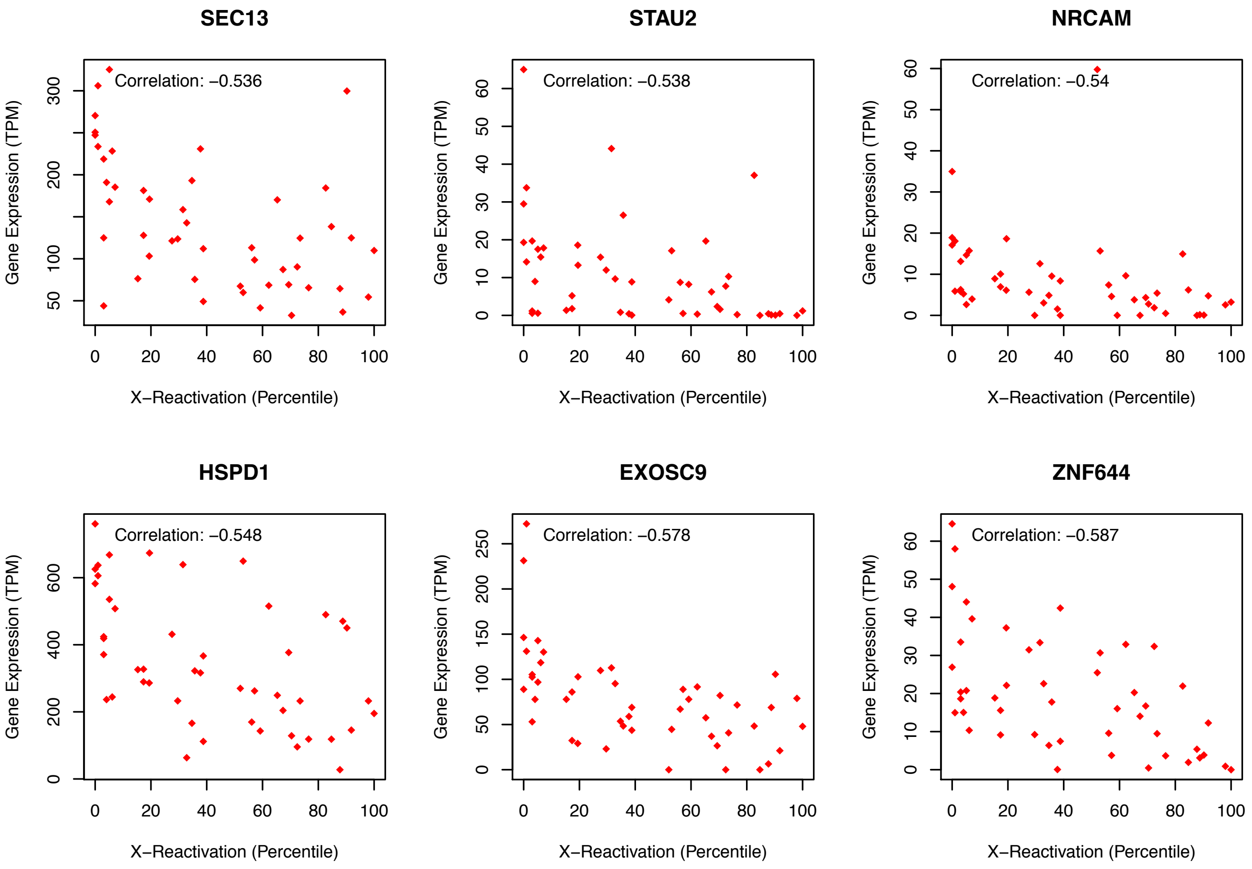
SEC13
Correlation: −0.536
STAU2
Correlation: −0.538
NRCAM
Correlation: −0.54
HSPD1
Correlation: −0.548
EXOSC9
Correlation: −0.578
ZNF644
Correlation: −0.587
Gene Expression (TPM)
X−Reactivation (Percentile)

Supplementary Figure 7

Autosomal genes most correlated and anti-correlated with germ cells ranked by X-reactivation.

Each plot shows gene expression of individual autosomal genes (transcripts per million, TPM) in germ cells ranked by X-reactivation status (see Fig. 4e). Spearman correlation in shown on the top of each graph. Positive correlated genes are shown in black dots and anti-correlated are shown in red dots.

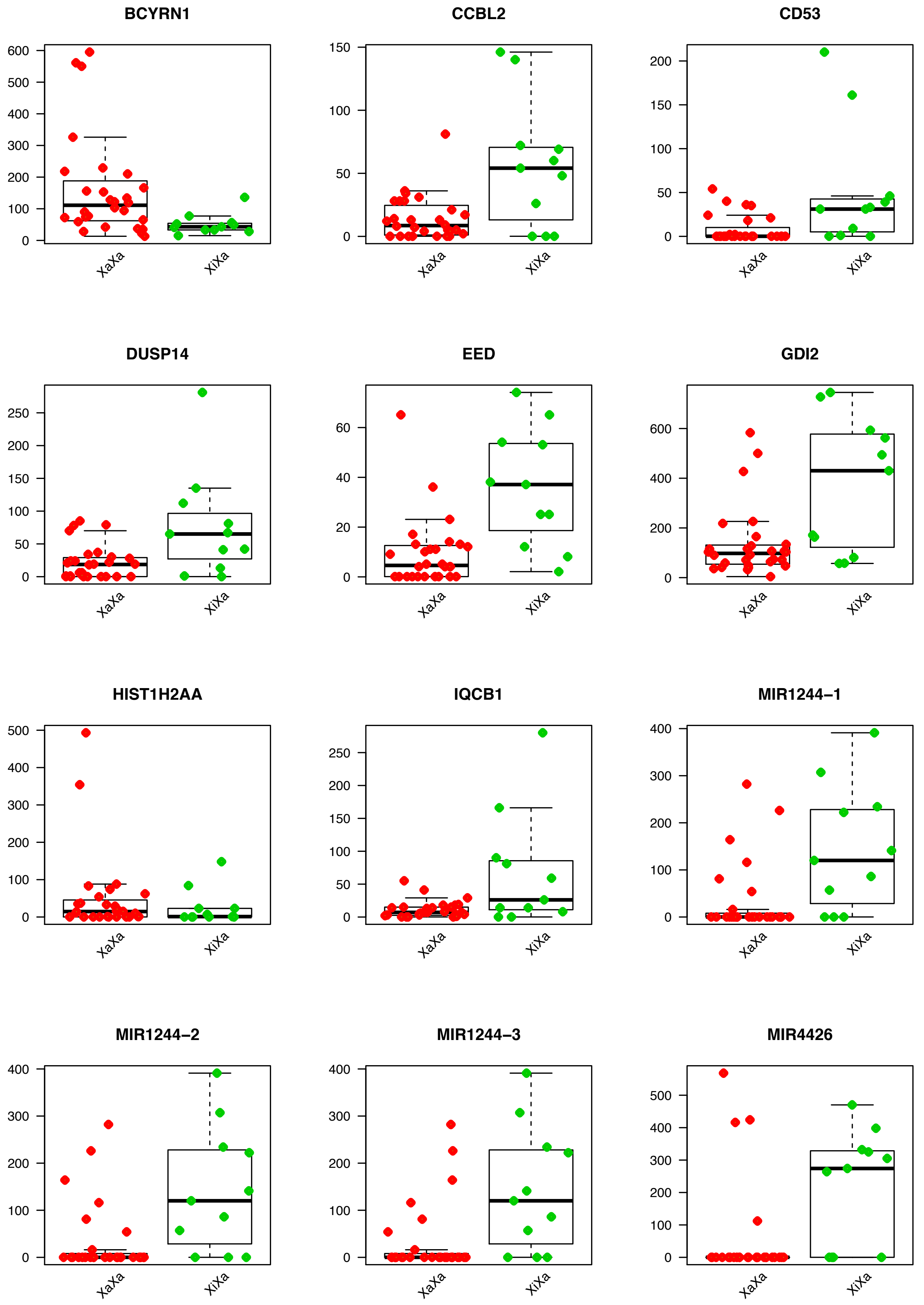
BCYRN1
600
500
400
300
200
100
0
XaXa
XiXa
CCBL2
150
100
50
0
XaXa
XiXa
CD53
200
150
100
50
0
XaXa
XiXa
DUSP14
250
200
150
100
50
0
XaXa
XiXa
EED
60
40
20
0
XaXa
XiXa
GDI2
600
400
200
0
XaXa
XiXa
HIST1H2AA
500
400
300
200
100
0
XaXa
XiXa
IQCB1
250
200
150
100
50
0
XaXa
XiXa
MIR1244−1
400
300
200
100
0
XaXa
XiXa
MIR1244−2
400
300
200
100
0
XaXa
XiXa
MIR1244−3
400
300
200
100
0
XaXa
XiXa
MIR4426
500
400
300
200
100
0
XaXa
XiXa

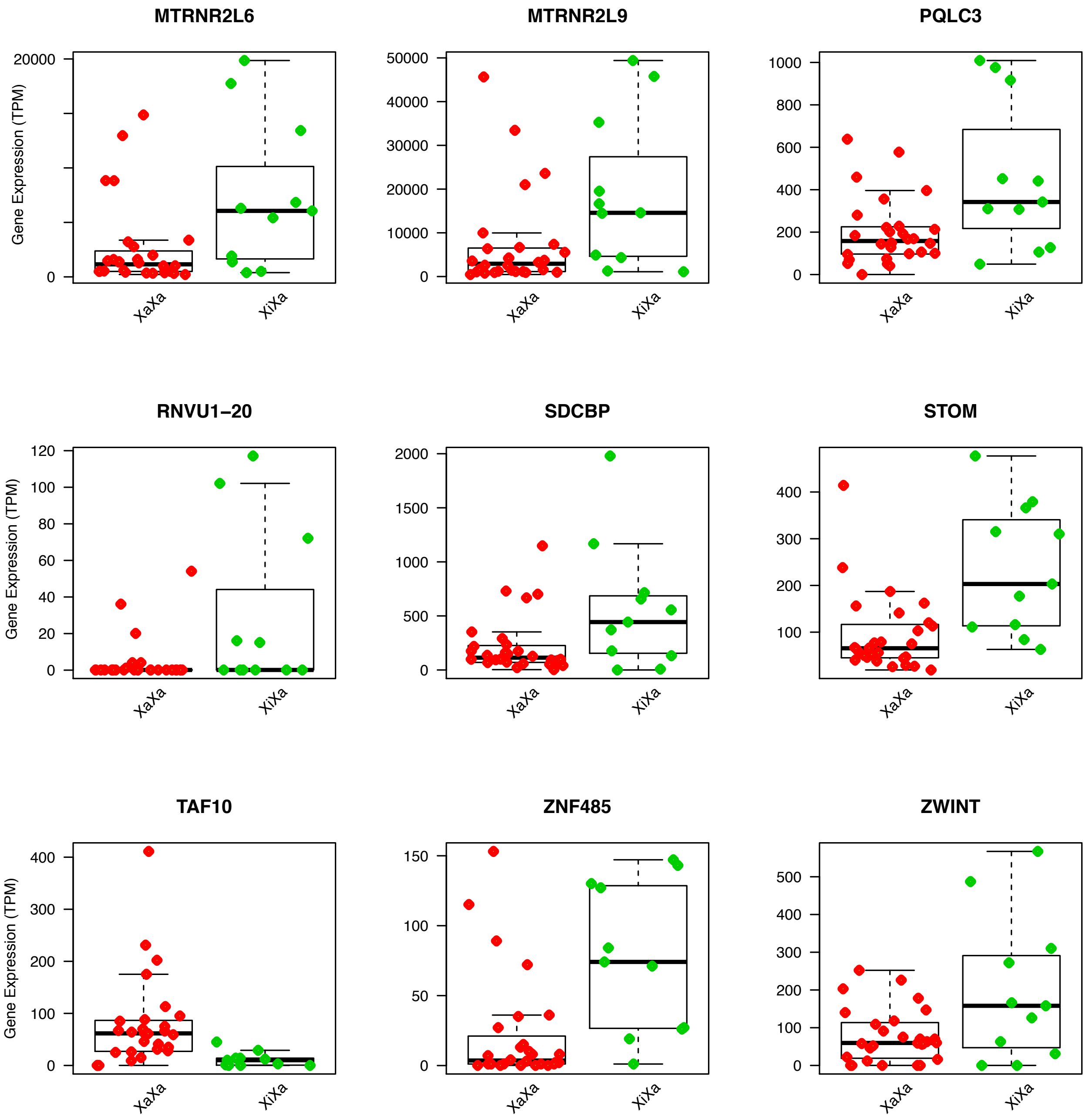
MTRNR2L6
MTRNR2L9
PQLC3
RNVU1−20
SDCBP
STOM
TAF10
ZNF485
ZWINT
Gene Expression (TPM)
XaXa
XiXa
20000
0
50000
40000
30000
20000
10000
1000
800
600
400
200
120
100
80
60
40
20
2000
1500
1000
500
300
150
50

Supplementary Figure 8
Differentially expressed genes between XaXi PGCs and XaXa PGCs.
Each plot shows gene expression of individual differentially expressed genes (transcripts per million, TPM) in XaXi PGCs and XaXa PGCs. Correlated with Fig. 4f.

Supplementary Tables

Supplementary Table 1
Sequences and coordinates of primers for targeted bisulfite sequencing.

Primer	Chr	Sequence	Strand	Start	End	Length (bp)
hKvDMR1_ICR_bis	11	FW 5' - 3' *GATGTGTATAAGAGACAG*GTATTTAGTTAATTYGTGTAGTAGTT<br>RV 5' - 3' *CGTGTGCTCTTCCGATCT*CAAAATCTTACTAAAAAACTCCCTA	-	2699936	2700121	236
hSNRPN_ICR_bis	15	FW 5' - 3' *GATGTGTATAAGAGACAG*AGGGAGTTGGGATTTTTGTATTG<br>RV 5' - 3' *CGTGTGCTCTTCCGATCT*CCCAAACTATCTCTTAAAAAAAACC	+	24954866	24955102	287
hPEG3_ICR_bis	19	FW 5' - 3' *GATGTGTATAAGAGACAG*GGTGTAGAAGTTTGGGTAGTTG<br>RV 5' - 3' *CGTGTGCTCTTCCGATCT*CTCACCTCACCTCAATACTAC	-	56840577	56840729	203
hMEG3_ICR_bis	14	FW 5' - 3' *GATGTGTATAAGAGACAG*GATTTTTTTTATATATTGTGTTTG<br>RV 5' - 3' *CGTGTGCTCTTCCGATCT*CTCATTTCTCTAAAAATAATTAACC	+	100825818	100826037	270
hH19_ICR_bis	11	FW 5' - 3' *GATGTGTATAAGAGACAG*TATGGGTATTTTTGGAGGTTTTTT<br>RV 5' - 3' *CGTGTGCTCTTCCGATCT*AATCCCAAACCATAACACTAAAAC	-	1999754	2000063	360
hH19_TSS_bis	11	FW 5' - 3' *GATGTGTATAAGAGACAG*GGAGTTGGTGGGTAGGGTTTGA<br>RV 5' - 3' *CGTGTGCTCTTCCGATCT*AACCACCCCTATACTCCCTCCC	-	1997161	1997490	380

Sequence in italics/underlined were adaptors for sequencing
Abbreviations: Chr, chromosome; bp, base pairs

Supplementary References

1. Baran Y, *et al.* The landscape of genomic imprinting across diverse adult human tissues. *Genome research* **25**, 927-936 (2015).
2. Guo F, *et al.* The Transcriptome and DNA Methylome Landscapes of Human Primordial Germ Cells. *Cell* **161**, 1437-1452 (2015).
