# Supplementary material for: Parental haplotype-specific single-cell transcriptomics reveal incomplete epigenetic reprogramming in human female germ cells
Source: Nat Commun. 2018 May 14;9:1873. doi: 10.1038/s41467-018-04215-7 (PMC5951918; doi:10.1038/s41467-018-04215-7)
Supplement: Supplementary file 3 — Description of Additional Supplementary Files [file 41467_2018_4215_MOESM3_ESM.pdf]

Description of Additional Supplementary Files

File name: Supplementary Data 1
Description: Enriched transcriptional signature in female germ cells.
Identity of (10 fold enriched) genes when cells were pooled either per germ cell stage (PGC, LGC, MGC) or per fetal age (4wk, 8-11wk, 14-17wk); including adrenal cells (AD) in the comparison.

File name: Supplementary Data 2
Description: List of confirmed imprinted genes, that were expressed in our dataset and contained SNPs, and their respective imprinting control region.

File name: Supplementary Data 3
Description: List of X-linked genes, that were expressed in our dataset and contained SNPs, and whether they have been reported to escape X chromosome inactivation.
